# Programmed suppression of oxidative phosphorylation and mitochondrial function by gestational alcohol exposure correlate with widespread increases in H3K9me2 that do not suppress transcription

**DOI:** 10.1186/s13072-021-00403-w

**Published:** 2021-06-15

**Authors:** Richard C. Chang, Kara N. Thomas, Nicole A. Mehta, Kylee J. Veazey, Scott E. Parnell, Michael C. Golding

**Affiliations:** 1 Department of Veterinary Physiology & Pharmacology, College of Veterinary Medicine and Biomedical Sciences, Texas A&M University, 4466 TAMU, College Station, TX 77843-4466 USA; 2 Present Address: Blumberg Laboratory, 4351 Natural Science II, University of California Irvine, Irvine, CA 92697 USA; 3 Present Address: Epizyme Inc, 400 Technology Square, Cambridge, MA 02139 USA; 4 Department of Cell Biology and Physiology, Bowles Center for Alcohol Studies, University of North Carolina, Chapel Hill, NC 27599-7178 USA

**Keywords:** Developmental programming, Fetal alcohol spectrum disorders (FASDs), Alcohol, H3K9me2, Epigenetic programming, Oxidative phosphorylation, mTORC2, Satb2, Chromatin looping, Birth defects

## Abstract

**Background:**

A critical question emerging in the field of developmental toxicology is whether alterations in chromatin structure induced by toxicant exposure control patterns of gene expression or, instead, are structural changes that are part of a nuclear stress response. Previously, we used a mouse model to conduct a three-way comparison between control offspring, alcohol-exposed but phenotypically normal animals, and alcohol-exposed offspring exhibiting craniofacial and central nervous system structural defects. In the cerebral cortex of animals exhibiting alcohol-induced dysgenesis, we identified a dramatic increase in the enrichment of dimethylated histone H3, lysine 9 (H3K9me2) within the regulatory regions of key developmental factors driving histogenesis in the brain. However, whether this change in chromatin structure is causally involved in the development of structural defects remains unknown.

**Results:**

Deep-sequencing analysis of the cortex transcriptome reveals that the emergence of alcohol-induced structural defects correlates with disruptions in the genetic pathways controlling oxidative phosphorylation and mitochondrial function. The majority of the affected pathways are downstream targets of the *mammalian target of rapamycin complex 2* (*mTORC2*), indicating that this stress-responsive complex plays a role in propagating the epigenetic memory of alcohol exposure through gestation. Importantly, transcriptional disruptions of the pathways regulating oxidative homeostasis correlate with the emergence of increased H3K9me2 across genic, repetitive, and non-transcribed regions of the genome. However, although associated with gene silencing, none of the candidate genes displaying increased H3K9me2 become transcriptionally repressed, nor do they exhibit increased markers of canonical heterochromatin. Similar to studies in *C. elegans*, disruptions in oxidative homeostasis induce the chromatin looping factor *SATB2*, but in mammals, this protein does not appear to drive increased H3K9me2 or altered patterns of gene expression.

**Conclusions:**

Our studies demonstrate that changes in H3K9me2 associate with alcohol-induced congenital defects, but that this epigenetic change does not correlate with transcriptional suppression. We speculate that the mobilization of SATB2 and increased enrichment of H3K9me2 may be components of a nuclear stress response that preserve chromatin integrity and interactions under prolonged oxidative stress. Further, we postulate that while this response may stabilize chromatin structure, it compromises the nuclear plasticity required for normal differentiation.

**Supplementary Information:**

The online version contains supplementary material available at 10.1186/s13072-021-00403-w.

## Introduction

Dysregulation of chromatin structure underlies several neurodevelopmental disorders and cognitive impairments [1]. However, emerging evidence reveals that histone H3 lysine 9 (H3K9), in particular, serves as a major hub in chromatin-based regulatory mechanisms important to central nervous system development and function. For example, disruption of acetylation on H3K9 leads to anxiety-related behavioral abnormalities [2–4] and blocking H3K9 deacetylation through pharmacological HDAC inhibitors serves as a powerful tool to counteract neurodegeneration-related cognitive decline [5, 6]. Similarly, inhibition of H3K9 methylation improves memory in the aged hippocampus [7, 8] and pharmacological inhibition of H3K9 methyltransferase activity restores alcohol-induced deficits in learning and memory [9–11]. Therefore, understanding the mechanisms driving the post-translational modifications of H3K9 is crucial to understanding central nervous system development and function.

We recently conducted a three-way comparison of chromatin structure between control offspring, alcohol-exposed but phenotypically normal animals (unaffected), and alcohol-exposed offspring exhibiting craniofacial and central nervous system structural defects (affected). In these studies, we focused on identifying changes in chromatin structure within the regulatory regions of genes directing the patterning of the brain that distinguish alcohol-exposed affected versus unaffected individuals. We identified a dramatic and widespread increase in the enrichment of dimethylated histone H3, lysine 9 (H3K9me2) in animals exhibiting alcohol-induced structural defects. At the same time, offspring in the alcohol-exposed unaffected group remained identical to the controls [12]. Why regulatory regions within affected tissues become enriched for H3K9me2 and whether this change in chromatin structure is a passive symptom of dysgenesis or causally involved in the development of structural defects remains unknown.

While exposure to drugs of abuse, including cocaine, morphine, and alcohol, all increase global acetylation and influence H3K9 methylation, these changes in chromatin structure scarcely correlate with altered gene expression, especially on a genome-wide scale [13, 14]. In contrast, each of these toxicants also associates with the development of oxidative stress, which induces protective alterations in chromatin structure [15, 16]. Recent work in the nematode *Caenorhabditis elegans* indicates that changes in chromatin structure, increased H3K9me2 specifically, constitute an integral component of the metabolic stress response [17]. Further, hypoxic stress can induce G9a-dependent increases in H3K9me2 across a range of cultured mammalian cells [18]. Therefore, the increased methylation of H3K9 observed in these studies and our model may be a component of the cellular stress response, independent of programs controlling transcriptional regulation.

One of the critical emerging questions in the field of exposure biology is whether toxicant-induced changes in chromatin structure are causal in altering gene expression or are symptoms of exposure tied to transcription-independent nuclear changes promoting genome stability. Although H3K9me2-enriched regions of the genome associate with gene silencing, heterochromatin also protects chromosome integrity during cellular stress [19]. Further, recent studies indicate that increased H3K9 methylation and formation of heterochromatin-like domains do not necessarily result in widespread gene repression [20]. Work in multiple organisms, including *C. elegans*, have demonstrated that the effects of early-life mitochondrial dysfunction and oxidative stress persist throughout the lifespan [21, 22]. Patterns of H3K9 methylation transmit through the cell cycle, and therefore, may represent a heritable memory of the larger nuclear organization induced by early-life exposures [23–26]. However, whether stress-induced H3K9 methylation directly influences patterns of gene expression remains unanswered.

We hypothesized that the increased Histone H3 Lysine 9 dimethylation observed in alcohol-exposed tissues exhibiting dysgenesis represent a core element in a conserved genome-wide defense mechanism, one that may not directly influence gene transcription. Using deep-sequencing, we find tissue-specific disruptions in the genetic pathways controlling oxidative phosphorylation and mitochondrial function associate with widespread increases in H3K9me2. This increased H3K9me2 does not correlate with transcriptional suppression, appears in a non-transcribed region of the genome, and does not associate with increased markers of canonical heterochromatin. Similar to studies in worms, we also observe the induction of the mammalian homolog of the *C. elegans* chromatin looping protein DVE-1 (SATB2) [17]. Importantly, our studies also implicate the mammalian target of rapamycin complex 2 (mTORC2) as one of the main drivers of this response, suggesting therapeutic interventions targeting this pathway may rescue alcohol-induced central nervous system patterning defects.

## Materials and methods

### Ethanol treatments and tissue collection

All animal procedures were approved and conducted in accordance with the Institutional Animal Care and Use Committee at the University of North Carolina. This study utilized C57BL/6J mice (RRID:IMSR_JAX:000664). We administered intraperitoneal injections of EtOH (2.9 g/kg, cat# E7023; Millipore-Sigma) to pregnant dams on gestational day 7 (GD7), as described previously [27]. Control dams were injected with a comparable volume of lactated Ringer’s solution. We terminated pregnancies on gestational day 17 (GD17) and then scored the offspring for ocular and cortical patterning defects using a previously described system [28, 29]. After assessment, we dissected the left and right cortices, cerebellum and heart, then immediately flash froze samples using liquid nitrogen.

### DNA and RNA isolation, and sequencing analysis

We isolated total RNA from the GD17 cortex using the RNeasy Plus Mini Kit (catalog # 74134, Qiagen) according to the manufacturer’s instructions. Before RNASeq library preparation, we randomized samples and generated sequencing libraries using the TruSeq RNA Sample Preparation kit (catalog # RS-122-2001, Illumina). We sequenced pooled samples using an Illumina HiSeq 2500 at Whitehead Genomic Services (Cambridge, MA). The sequencing data were demultiplexed, aligned using STAR with default parameters [30], and referenced against the *Mus musculus* genome (UCSC version mm10).

### RNA deep sequencing data analysis, selection of candidate mRNAs, and functional analysis

After obtaining 50-bp length paired-end reads, we used Bowtie and Tophat to align them into transcripts based on the Mouse Reference Genome (UCSC version mm10). We then used the Cufflinks suite to measure the relative abundance of each transcript. We quantified gene expression levels as the number of reads mapping to a gene divided by the gene length in kilobases and the total number of mapped reads in millions, and designated outputs as fragments per kilobase of exon per million fragments mapped (FPKM). To select differentially expressed transcripts, we generated volcano plots measuring statistical significance and magnitude of fold-change based on the log2 fold-change (*X*-axis) and − log10 *p*-value from the Cufflinks suite (*Y*-axis). We selected differentially expressed mRNAs based on a linear *p*-value cut off of 0.05, which was considered significant and highlighted by colored dots in the volcano plot. We conducted the Causal Network Analysis and Biological Pathway Enrichment analysis using the Ingenuity Pathway Analysis (IPA) software suite (QIAGEN Inc.) [31].

### Neural stem cell culture and EtOH exposure

Derivation of primary mouse fetal cerebral cortical neuroepithelial stem cells is described here [32]. We cultured free-floating neurospheres in T75 flasks containing a 50%/50% mixture by volume of Neurobasal media (Cat# 21103-049; Invitrogen) and DMEM F-12 (Cat# 11320-033; Invitrogen). This medium was supplemented with the N2-supplement (Cat# 17502-048; Invitrogen), B27 supplement (Cat# 17504-044; Invitrogen), 0.05% TC grade BSA in PBS (Cat# A1933 Sigma), 2 mM l-glutamine (Cat# 25030-081; Invitrogen), 1× penicillin/streptomycin (Cat# 15140-122; Invitrogen), 20 µg/mL FGF basic (Cat# PMG0034; Invitrogen), 20 µg/mL EGF (Cat# PHG0311; Invitrogen), and 0.85 units/mL heparin (Cat# H3149; Sigma). We cultured cells in medium containing either 160 mg/dL, 240 mg/dL, or 0 mg/dL (control) EtOH (cat# E7023; Millipore-Sigma). We cultured neurospheres in treated medium for 72 h, and collected samples for ChIP and RNA analysis after a 4-day recovery phase with no EtOH.

### Chromatin immunoprecipitation (ChIP)

We thawed the cortices derived from a single brain and filtered the tissues into a single-cell suspension using gentle mechanical dissociation. We washed cells twice with PBS containing protease inhibitor cocktail (Cat# 78437; Thermo Scientific) and re-suspended them in medium (DMEM F-12 Cat# 11320-033; Invitrogen) containing 0.1 volume crosslinking solution [33]. We carried out chromatin immunoprecipitation reactions following the previously published protocol [34]. The specific antibodies used in this study include anti-H3K9me2 (Cat# 39239; Active Motif, RRID:AB_2793199), antiH3K9me3 (Cat# 05-1242; Millipore-Sigma, RRID:AB_1587136), antiH4K20me3 (Cat# 91107; Active Motif, RRID:AB_2793777), anti-SATB2 (Cat# ab34735; Abcam, RRID:AB_2301417) and the negative IgG control (Cat# SC-2027; Santa Cruz, RRID:AB_737197). We used antibodies for modified histones and the IgG control at a concentration of 1 µg/ChIP reaction and 4 µg/ChIP reaction for SATB2. We purified precipitated DNA using the QIAquick PCR Purification Kit (catalog # 28106, Qiagen) and assayed the enrichment of the indicated sequences using quantitative PCR. We performed qPCR using the Dynamo Flash supermix (Cat# F-415XL; Thermo Scientific) on a Bio-Rad CFX384 Touch PCR system. Primer sequences are listed in Additional file 1.

### Analysis of gene expression

We isolated total RNA from the GD17 fetal cerebellum and heart using the AllPrep DNA/RNA/Protein Mini Kit (Catalog No. 80004; Qiagen) according to the manufacturer’s instructions. We then seeded 1 μg of RNA into a reverse transcription reaction using the High Capacity cDNA Reverse Transcription Kit (Catalog No. 4368814; Thermo Fisher) following the recommended protocol. We assayed gene expression using the DyNamo Flash SYBR qPCR kit (Catalog No. F-415; Thermo Fisher) on a Bio-Rad CFX384 Touch PCR system. Primer sequences are listed in Additional file 1. To normalize gene expression levels, we assayed the expression of the reference genes Glyceraldehyde 3-phosphate dehydrogenasetyrosine (*Gapdh*), 3-monooxygenase/tryptophan 5-monooxygenase activation protein zeta (*Ywhaz*), and H2A Histone family member Z (*H2afz*). For studies in the fetal cortex, we used *Gapdh* and *Ywhaz*. For studies in the cerebellum and heart, we replaced *Gapdh* with *H2afz* due to increased stability [35].

### Western immunoblot analysis

We isolated protein from the GD17 fetal cortex using the AllPrep DNA/RNA/Protein Mini Kit (Catalog No. 80004; Qiagen) according to the manufacturer’s instructions. We then separated 5–10 µg of protein on 10–15% sodium dodecyl sulfate-polyacrylamide gels and transferred proteins to nitrocellulose membranes. The primary antibodies we used in this study are: anti-H3K9me2 (Cat# 39239; Active Motif, RRID:AB_2793199), anti-pan histone H3 (Cat# ab1791; Abcam, RRID:AB_302613), anti-SATB2 (Cat# ab34735; Abcam, RRID:AB_2301417) and anti-Tubulin (Cat# ab40742; Abcam, RRID:AB_880625). We visualized blots using secondary antibodies conjugated to horseradish peroxidase (catalog# sc-2004; RRID:AB_631746; Santa Cruz Biotechnology, Santa Cruz, CA, USA) and an enhanced chemiluminescence detection system (Pierce, Rockford, IL, USA). We calculated relative levels of H3K9me2 as a ratio to total histone H3, and relative levels of SATB2 as a ratio to Tubulin. We quantified band intensities by densitometry using ImageJ (RRID:SCR_003070; National Institutes of Health, Bethesda, MD, USA). Each experimental group contains protein extracts derived from 5 control, 3 unaffected, 4 affected animals.

### Statistical analysis

For RT-qPCR analysis of gene expression, we imported the replicate cycle threshold (Ct) values for each transcript into Excel and normalized expression to the geometric mean of two validated reference genes [35]. We then used the −∆∆CT method [36] to calculate the relative fold-change for each biological replicate. For studies using ChIP, we calculated the enrichment of each sequence using the published formula [37]. After collating datasets in Excel, we then input the calculated values into the statistical analysis program GraphPad Prism 8 (RRID:SCR_002798; GraphPad Software, Inc., La Jolla, CA, USA) and set statistical significance set at alpha = 0.05. We then verified all datasets for normality using the Brown–Forsythe test. For the analysis of ChIP and RT-qPCR data, we conducted a one-way analysis of variance followed by an uncorrected Fisher’s LSD post hoc test. Data presented are mean ± standard error of the mean.

## Results

### Transcriptome analysis of the alcohol-exposed, affected fetal cortex reveals deregulation of oxidative phosphorylation and mitochondrial pathways

**Fig. 1:**
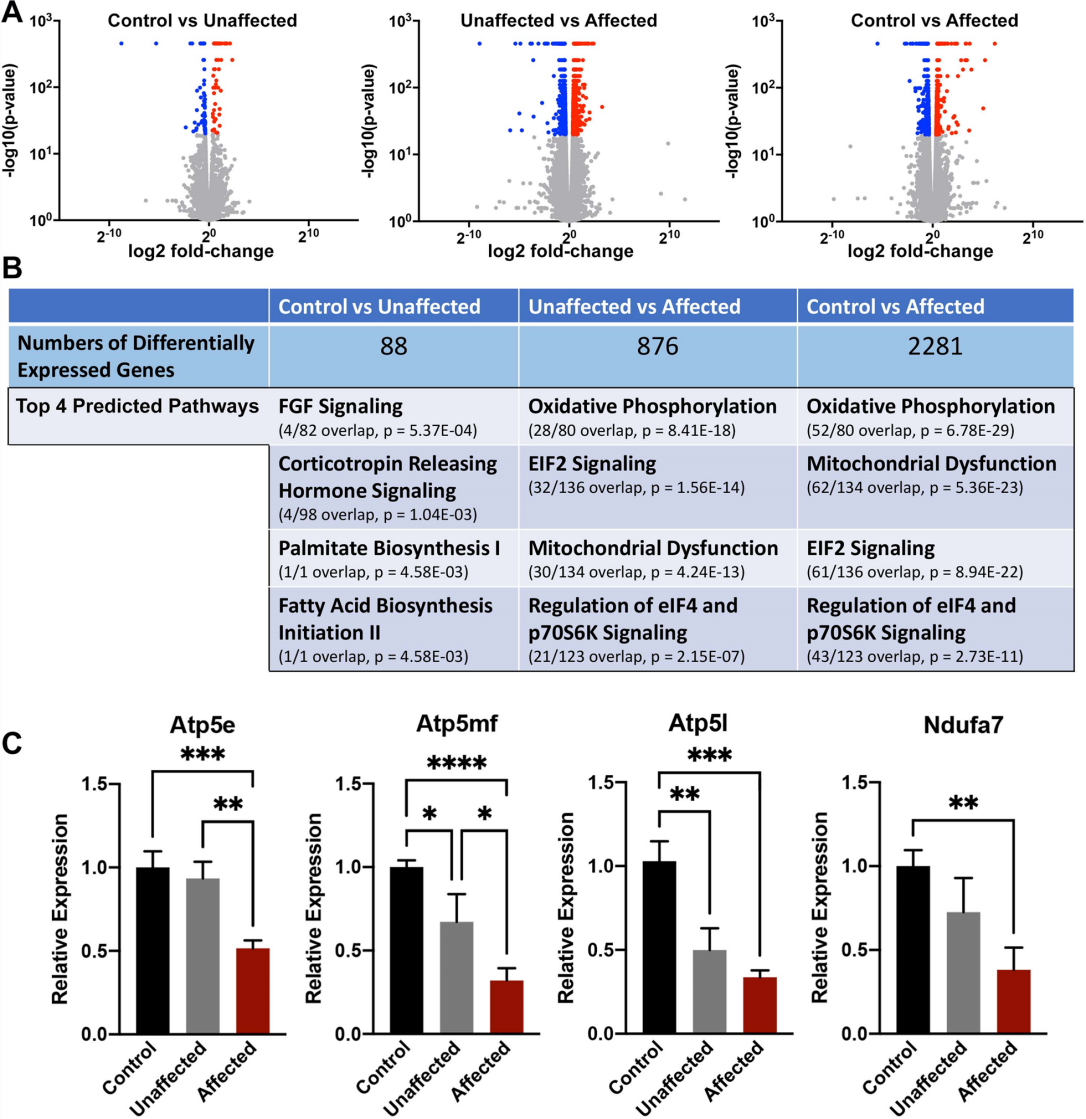
Comparison of transcriptomes between control, alcohol-exposed unaffected, and affected offspring. Pregnant dams were injected with 2.9 g/kg EtOH on gestational day-seven, and on gestational day-17, individual embryos were sorted based on the presence of ocular and cortical patterning defects. We dissected the fetal cortex and analyzed the transcriptome using deep-sequencing. **A** Volcano plots comparing offspring between the indicated groups (*n* = 3). **B** The top four biological categories identified using Ingenuity Pathway Analysis. **C** Validation of identified candidate genes within the Oxidative Phosphorylation, Mitochondrial Dysfunction, and EIF2 Signaling pathways. Analysis of gene expression carried out using RT-qPCR. Gene expression was normalized to transcripts encoding *Gapdh* and *Ywhaz* (*n* = 7). Error bars represent the SEM, *P < 0.05, **P < 0.01, ***P < 0.001, ****P < 0.0001

To identify transcriptional differences distinguishing affected fetuses from unaffected offspring, we returned to our previously published, early gestational binge model of maternal alcohol exposure [12]. Here, dams were intraperitoneally administered injections of either vehicle or alcohol on gestational day-7. On gestational day-17 (GD17), stage-matched control and alcohol-exposed fetuses were dissected and scored for both ocular and craniofacial congenital defects. After determining the sex of each fetus using PCR analysis targeting the Y-chromosome, we found that the majority of affected samples (13/17; 76%) were female. We then isolated RNA from the GD17 fetal cortex of three (*n* = 3) control, EtOH-exposed unaffected, and EtOH-exposed affected offspring and conducted deep-sequencing analysis of the cortex transcriptome. Our sequencing analyses identified a small number (88) of differentially expressed transcripts between the control and unaffected samples. In contrast, comparisons between control and affected, as well as unaffected and affected, identified 876 (412 downregulated, 522 upregulated) and 2281 (1432 downregulated, 1041 upregulated) differentially expressed genes, respectively (Fig. 1A, B).

The top three molecular pathways identified by Ingenuity Pathway Analysis [31] between unaffected: affected and control: affected comparisons were Oxidative Phosphorylation, Mitochondrial Dysfunction and *EIF2* Signaling (Fig. 1B), all of which converge on the regulation of cellular respiration or the oxidative stress response [17, 38]. We validated the differential expression of the top genes in the Oxidative Phosphorylation and Mitochondrial Dysfunction pathways using RT-qPCR (Fig. 1C). These assays confirmed the downregulation of all candidates in the EtOH-exposed affected offspring. With a larger sample size, we also observed some differential expression of select candidate genes in the EtOH-exposed unaffected group. However, these samples tended to exhibit a lower fold-change than the affected offspring.

### Alcohol-induced disruption of genes controlling cellular respiration and oxidative stress is tissue specific

**Fig. 2:**
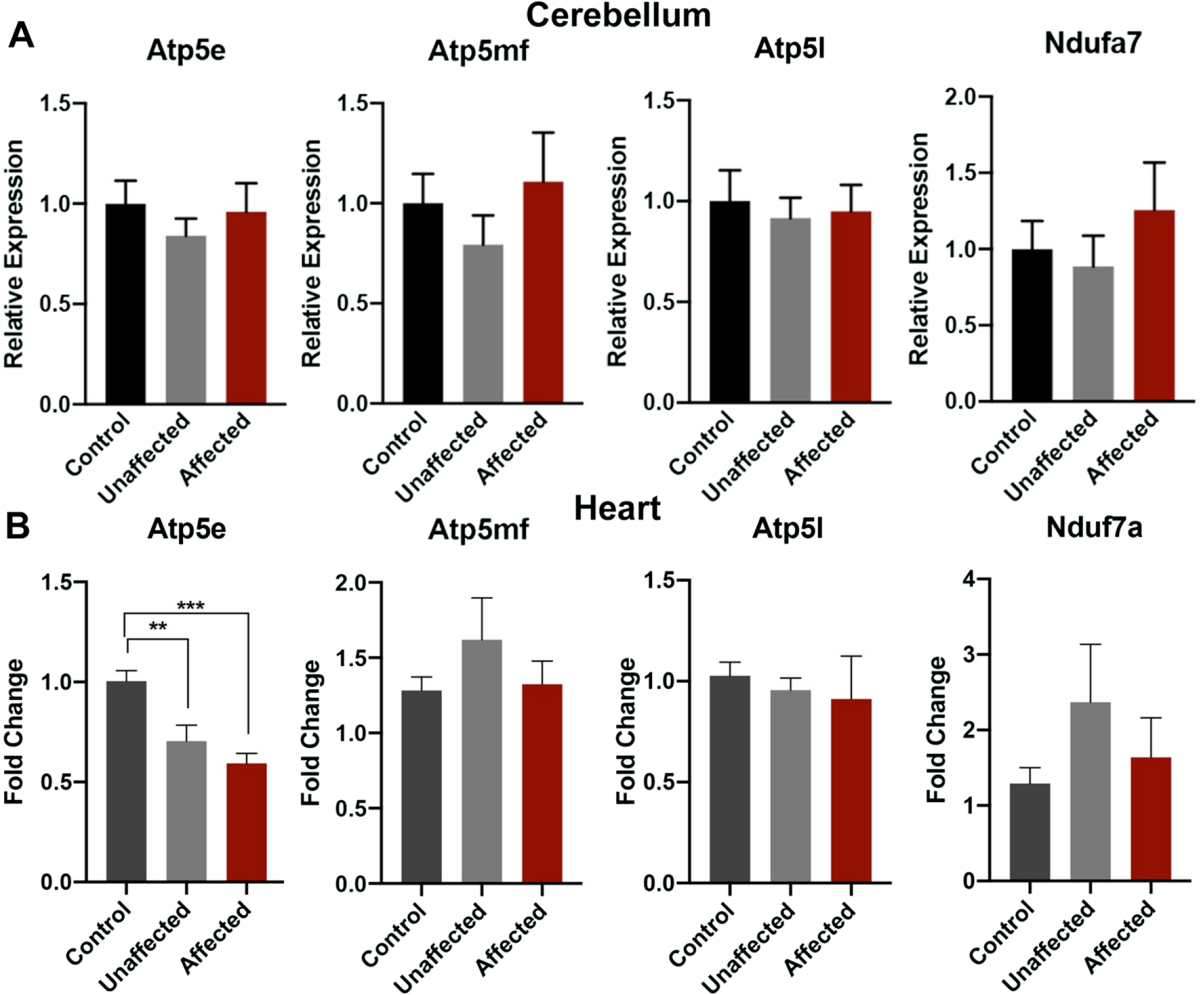
Analysis of candidate gene expression in the cerebellum and heart. Examination of candidate genes within the Oxidative Phosphorylation, Mitochondrial Dysfunction, and EIF2 Signaling pathways using RNA samples derived from morphologically normal **A** cerebellum and **B** heart. Tissues were isolated from control, alcohol-exposed but phenotypically normal offspring, and alcohol-exposed affected offspring, exhibiting ocular and cortical patterning defects. Analysis of gene expression conducted using RT-qPCR, normalized to transcripts encoding *H2Afz* and *Ywhaz*. (*n* = 8 Control, 9 Unaffected, 7 Affected). Error bars represent the SEM, *P < 0.05, **P < 0.01, ***P < 0.001

In our model, gestational day-7 exposures predominantly impact cortical development, while sparing the cerebellum [39]. In contrast, exposures on gestational day-8 induce significant cerebellar-specific reductions in volume, indicating stage-specific sensitivities of these subregions [40]. To determine if the transcriptional signature identified in the cortices of EtOH-exposed affected offspring is present in morphologically normal tissues, we assayed the abundance of transcripts encoding the above candidate genes within the cerebellum. We also examined the heart, as the development of this organ is exquisitely sensitive to oxidative stress [41]. As shown in Fig. 2A, we did not observe any differences in candidate gene expression in the cerebellum. At the same time, while hearts exhibited downregulation of *Atp5e* in tissues isolated from both unaffected and affected offspring, we did not detect differences in any other candidate gene (Fig. 2B). From these data, we conclude that the programmed suppression of the genetic pathways regulating Oxidative Phosphorylation, Mitochondrial Dysfunction, and *EIF2* Signaling is unique to the cortex of EtOH-exposed affected brains.

### Affected tissues display upregulation of mTORC2 and correlative increases in histone H3 lysine 9 dimethylation that do not associate with transcriptional repression

Out of the 109 identified candidate genes in the Oxidative Phosphorylation and Mitochondrial Dysfunction pathways, 105 are targets of the *mammalian target of rapamycin complex 2* (*mTORC2*) (**p-value = 9.19E−42**). Consistent with these findings, we observed upregulation of multiple *mTORC2* complex members in the alcohol-exposed affected offspring (**p-value = 4.19E−09**) and downregulation of targets known to be negatively regulated by *mTORC2* [42]. In mammals, mTORC2 has an established role in directing epigenetic changes in gene function, leading to enduring alterations in oxidative respiration and metabolism [43]. However, how mTORC2 establishes and propagates this epigenetic memory is unknown. In yeast, the TOR2 complex is essential for establishing histone h3 lysine 9 dimethylation (H3K9me2) and promoting heterochromatin formation, stability, and spreading [44]. Further, two independent studies using cultured mammalian cells and *C. elegans* identified a link between oxidative stress, the nuclear lamina, and increased global enrichment of H3K9me2 [17, 18].

**Fig. 3:**
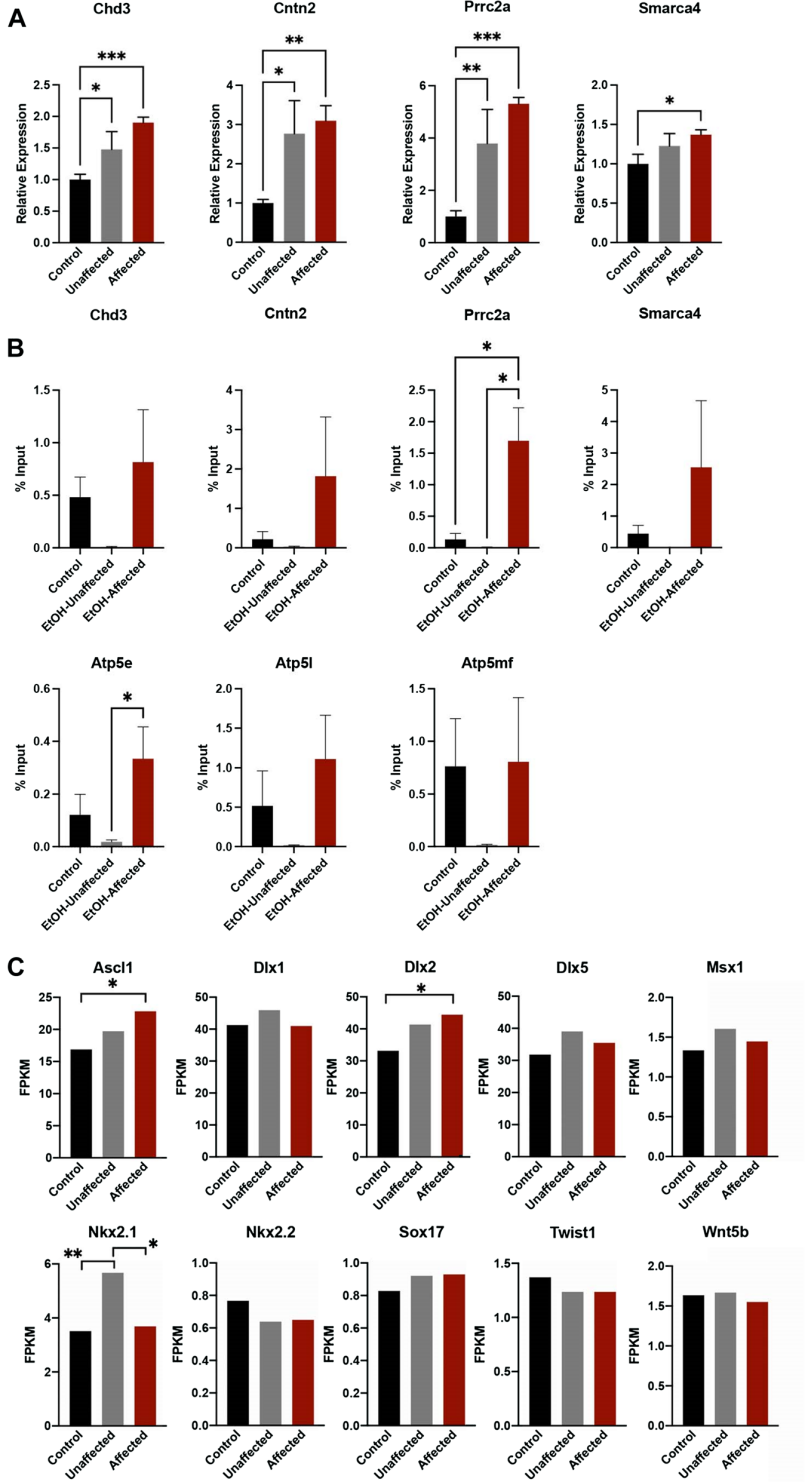
Increases in histone H3, lysine 9 dimethylation do not associate with candidate gene transcriptional silencing. **A** RT-qPCR validation of the top upregulated genes in the cortices of control, EtOH-exposed unaffected, and EtOH-exposed affected fetal mice. Gene expression was normalized to transcripts encoding Gapdh and Ywhaz. (*n* = 7). **B** Using chromatin immunoprecipitation qPCR (ChIP-qPCR), we assayed the enrichment of histone H3, lysine 9 dimethylation (H3K9me2) within the regulatory regions of upregulated and downregulated candidate genes. **C** Transcript levels of previously identified candidate genes exhibiting a > 500% increase in the enrichment of H3K9me2 within their regulatory regions. *P < 0.05, **P < 0.01, ***P < 0.001

Our previous experiments examining alcohol-induced epigenetic changes in the developing brain identified increased H3K9me2 in the promoter regions of several essential genes controlling cellular identity and proliferation [12]. To determine if the differentially expressed genes we identified here also display this change, we selected a cohort of downregulated (Fig. 1C) and upregulated (Fig. 3A) candidate genes and assayed the enrichment of H3K9me2 within their promoter regions. As seen in Fig. 3B, only the promoter region of *Prrc2a*, an upregulated gene, displayed increased H3K9me2 compared to the control and unaffected offspring. We then turned to our RNA-seq dataset to examine transcript levels of the previously identified candidate genes exhibiting increased H3K9me2. Despite showing dramatic increases in H3K9me2 enrichment [12], only Ascl1 and Dlx2 were differentially expressed, and similar to *Prrc2a*, both of these candidates also displayed increased expression (Fig. 3C).

**Fig. 4:**
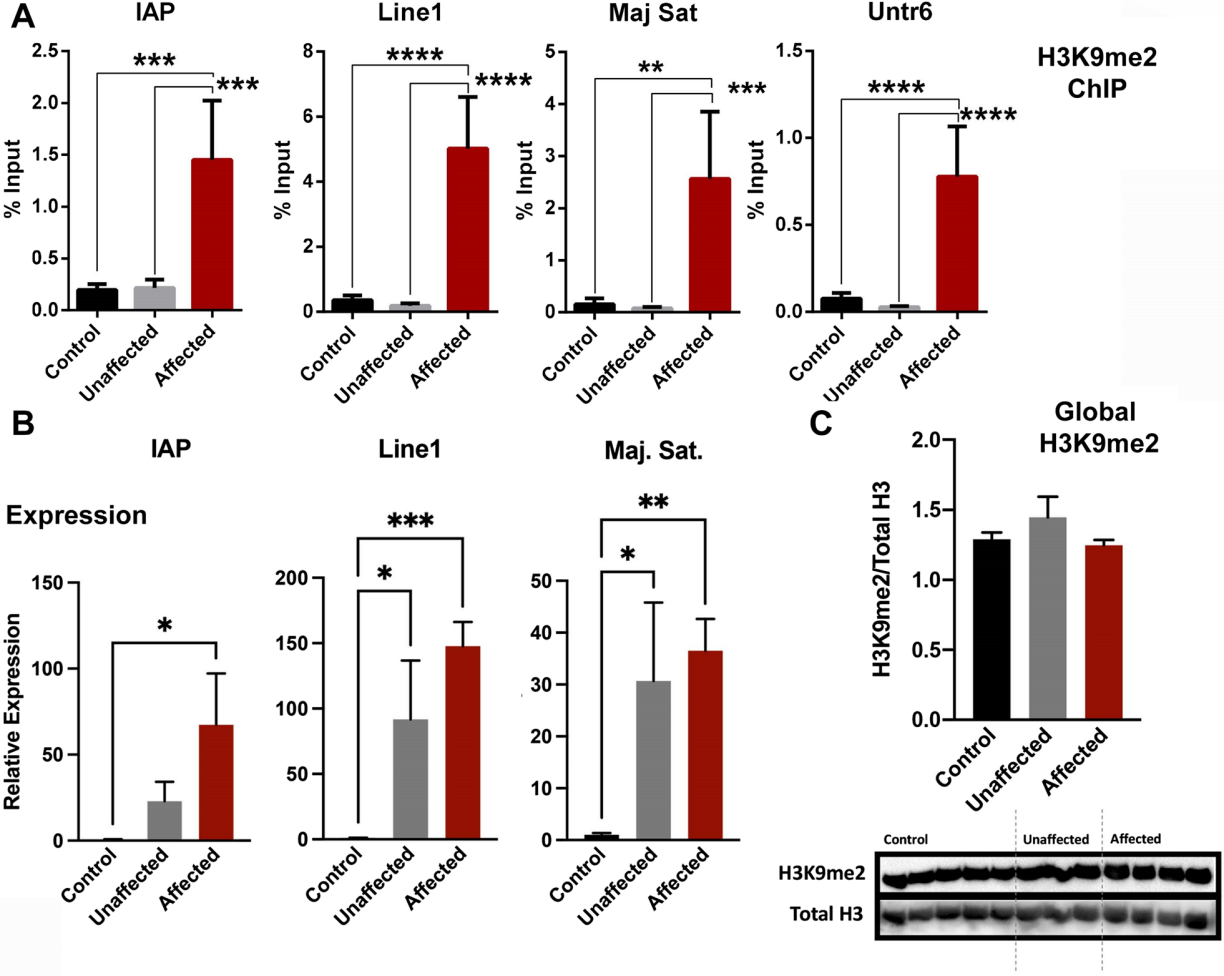
Widespread increases in histone H3, lysine 9 dimethylation does not associate with increased transcriptional suppression. **A** We assayed cellular extracts isolated from the fetal cortex of control, EtOH-exposed unaffected, and EtOH-exposed affected offspring for the enrichment of histone H3, lysine 9 dimethylation (H3K9me2) using chromatin immunoprecipitation (ChIP) followed by qPCR analysis. Our ChIP-qPCR primers assayed H3K9me2 within an untranscribed region of chromosome 6 (Untr6) and the indicated transposable elements. **B** RT-qPCR analysis of transcripts encoding the three families of transposable elements assayed above. Expression levels were normalized to transcripts encoding *Gapdh* and *Ywhaz*. **C** Analysis of global levels of H3K9me2 using western blotting. Representative western blot, with levels of H3K9me2 normalized to total histone H3 (*n* = 7). *P < 0.05, **P < 0.01, ***P < 0.001, ****P < 0.0001

To determine how widespread this increase in H3K9me2 is, we measured the enrichment of this post-translational modification within the regulatory regions of IAP, LINE1 elements, and major satellite class repeats between control, EtOH-exposed unaffected, and EtOH-exposed affected brain cortex. In addition, we also determined the enrichment of H3K9me2 in an untranscribed region of chromosome 6 (Untr6), which provides a measure of genomic background in ChIP assays examining transcription factor binding [45]. These experiments revealed increased enrichment of H3K9me2 in all three classes of repetitive DNA, as well as in the untranscribed region of chromosome 6 (Fig. 4A). Again, the increased H3K9me2 within repetitive sequences associated with increased transcription, not silencing (Fig. 4B). Unexpectedly, in EtOH-exposed unaffected cortices, we observed upregulation of LINE1 and major satellite class repeats, whereas IAP elements were only upregulated in EtOH-exposed affected samples. To determine if the enrichment in H3K9me2 reflects a global increase, we quantified H3K9me2 using western blotting and normalized measures to levels of total histone H3. As seen in Fig. 4C, we did not observe any differences in total cellular H3K9me2 between experimental groups. From these data, we conclude that EtOH-exposed affected tissues exhibit widespread increases in H3K9me2 that do not strictly associate with transcriptional suppression and are not reflective of a genome-wide increase in this post-translational modification.

### Alcohol-induced enrichment of H3K9me2 does not associate with the formation of canonical heterochromatin

**Fig. 5:**
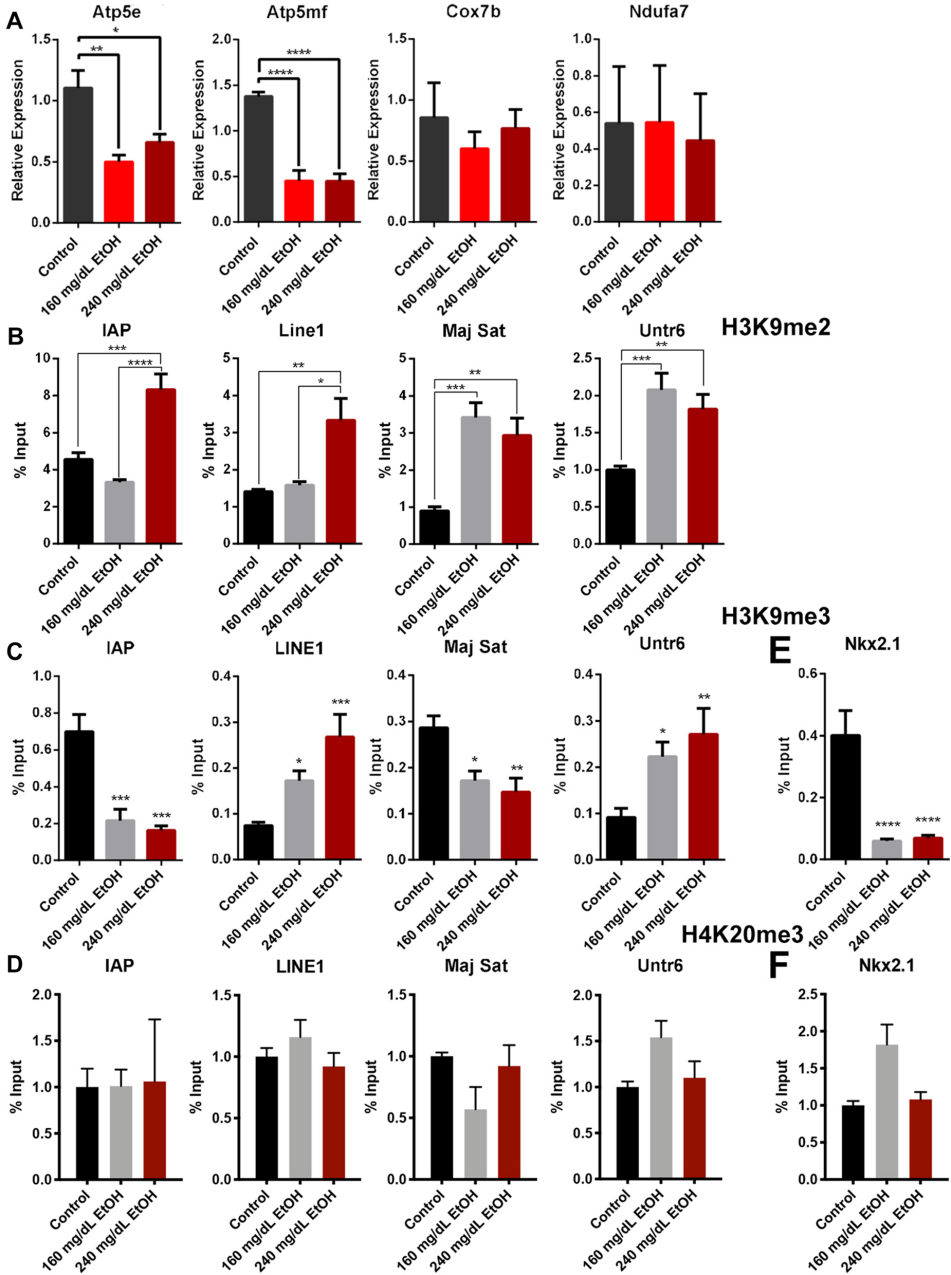
In vitro analysis of alcohol-induced changes in histone post-translational modifications. Primary fetal cerebral cortical neuroepithelial stem cells were cultured in the presence of 160 or 240 mg/dL EtOH for 3 days, followed by a 4-day recovery in medium lacking EtOH. **A** Examination of transcripts encoding candidate genes within the Oxidative Phosphorylation, Mitochondrial Dysfunction, and *EIF2* Signaling pathways in RNA samples isolated from treated neuroepithelial stem cells. Gene expression was normalized to transcripts encoding *Gapdh* and *Ywhaz* (*n* = 4). We assayed cellular extracts isolated from treated neuroepithelial stem cells for the enrichment of **B** histone h3, lysine 9 dimethylation (H3K9me2) **C** histone h3, lysine 9 trimethylation (H3K9me3), and **D** histone h4 lysine 20 trimethylation (H3K20me3). ChIP-qPCR primers assayed the enrichment of the post-translational modifications within an untranscribed region of chromosome 6 (Untr6) and the indicated transposable elements. Enrichment of **E** H3K9me3 and **F** H4K20 within the regulatory region of *Nkx2.1*. For ChIP-qPCR analysis *n* = 4. Error bars represent the SEM, *P < 0.05, **P < 0.01, ***P < 0.001, ****P < 0.0001

Di-methylation and tri-methylation of lysine nine are recognized components of heterochromatin that, along with *HP1* and histone H4 lysine 20 methylation, play an essential role in transcriptional silencing and genome stability [19]. To determine if alcohol selectively impacts only the dimethylated state of H3K9, we returned to our previously published primary neurosphere model of exposure [12, 13]. Here, we exposed cultured cells to 0, 160, and 240 mg/dL of alcohol for 3 days, then passaged exposed cells without alcohol for a 4-day recovery phase. Importantly, our cell culture experiments largely reproduced both the suppression of mTORC2 target genes (*Atp5e* and *Atp5mf*) and increases in H3K9me2 within repetitive sequences that we observed in our mouse model (Fig. 5A, B). Using ChIP, we next assayed the enrichment of trimethylated histone H3 lysine 9 (H3K9me3), as well as trimethylation of histone H4 lysine 20 (H4K20me3), which both co-localize during the formation of canonical heterochromatin [46]. These experiments revealed decreased H3K9me3 in both IAP and major satellite repeats, while LINE1 elements and the untranscribed region of chromosome 6 both exhibited increases (Fig. 5C). In contrast, we observed no differences in H4K20me3 across any repeat regions or Untr6 (Fig. 5D).

To expand our analysis into protein-coding genes, we assayed the promoter region of the homeobox gene *Nkx2.1*, which exhibited a dramatic increase in H3K9me2 both in vitro and in vivo [12]. The regulatory region of *Nkx2.1* displayed a 75% decrease in H3K9me3, while the enrichment of H4K20me3 was not significantly different between treatments (Fig. 5E, F). Although the cortices of alcohol-exposed animals exhibiting congenital defects consistently display increased H3K9 dimethylation, H3K9me3 shows region-specific changes, while we did not observe any significant impact on H4K20me3 enrichment. From these data, we conclude that the observed ethanol-induced increases in H3K9me2 do not associate with the formation of canonical heterochromatin.

### Increased expression SATB2 in alcohol-exposed affected tissues does not correlate with altered gene expression or increased H3K9me2

**Fig. 6:**
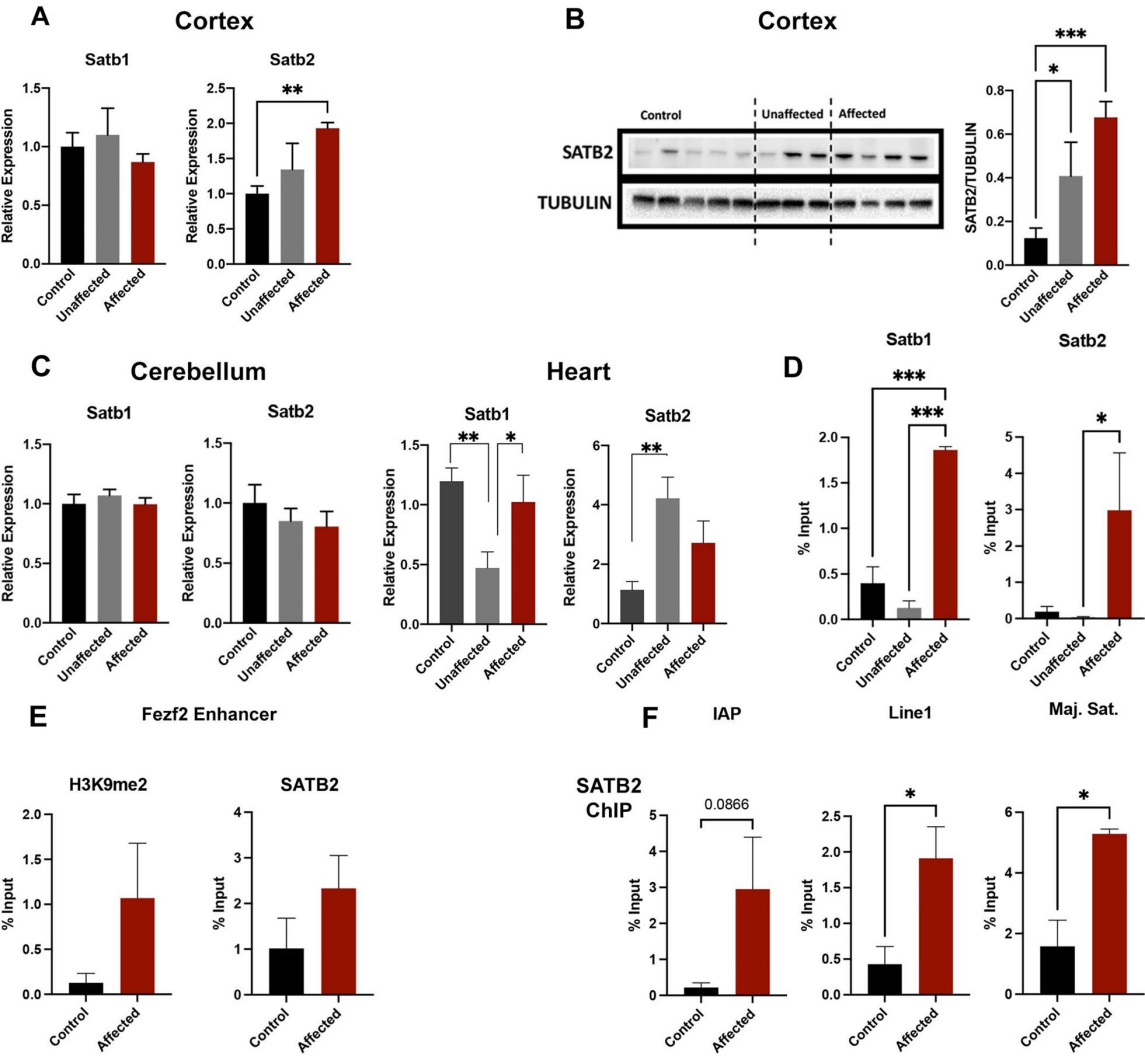
Increased expression of *Satb2* in the cortex of alcohol-exposed animals does not correlate with alterations in gene expression or increased H3K9me2. **A** Using RT-qPCR, we assayed levels of transcripts encoding the chromatin looping proteins *Satb1* and *Satb2* between control, alcohol-exposed but normal, and alcohol-exposed animals exhibiting structural defects. **B** Western blot analysis of SATB2 protein levels within the fetal cortex across treatment groups, with samples normalized to TUBULIN. **C** RT-qPCR analysis of *Satb1* and *Satb2* in the cerebellum and heart. **D** Using samples derived from the fetal cortex and ChIP-qPCR, we assayed the *Satb1* and *Satb2* promoters for the enrichment of H3K9me2. **E** As a positive control, we used ChIP-qPCR to determine the enrichment of H3K9me2 (left) and SATB2 (right) at the *Fezf2* upstream enhancer, a known SATB2 binding site [53]. **F** Using ChIP-qPCR, we determined SATB2 enrichment at the indicated transposable elements. For the cortex, gene expression was normalized to transcripts encoding *Gapdh* and *Ywhaz*, while in the cerebellum and heart, expression was normalized to the transcripts encoding *H2Afz* and *Ywhaz* (*n* = 8 Control, 9 Unaffected, 7 Affected). Error bars represent SEM, *P < 0.05, **P < 0.01

Previous studies using worms and mammalian cells demonstrate that mitochondrial dysfunction and oxidative stress induce global enrichment of H3K9me2 [17, 18]. In worms, upregulation, and nuclear localization of the chromatin looping protein DVE-1 precedes the global enrichment of H3K9me2, which together serve to stabilize chromatin interactions [17]. The mammalian homologs of DVE-1 are *SATB1* and *SATB2*, which also play major roles in organizing chromatin looping and stabilize interactions between gene enhancers and promoters [47–52]. Further, *SATB2* function is critical for proper development of the cerebral cortex and normal craniofacial patterning [50, 53]. To determine if the DVE-1 homologs *SATB1* and *SATB2* are upregulated in our model, we assayed their expression in the cortices of control, EtOH-exposed unaffected, and EtOH-exposed affected offspring. Consistent with studies in *c. elegans*, we observed an increase in transcripts encoding *Satb2* in the cortices of alcohol-exposed affected but not in control or alcohol-exposed unaffected offspring (Fig. 6A). Using Western blotting, we validated upregulation of *SATB2* in alcohol-exposed affected offspring (Fig. 6B). We also observed a significant, although more minor, magnitude increase of *SATB2* protein in cortices derived from EtOH-exposed unaffected offspring. Similar to candidate genes in the pathways regulating Oxidative Phosphorylation and Mitochondrial Dysfunction, we only identified *Satb2* upregulation in the cortex and not the heart or cerebellum of EtOH-exposed, affected animals (Fig. 6C). We then assayed enrichment of H3K9me2 in the *Satb1* and *Satb2* regulatory regions. The *Satb1* promoter displayed increased H3K9me2, and although the *Satb2* promoter trended toward an increase, this did not reach statistical significance (*p* = 0.1) and was only different from samples derived from EtOH-exposed unaffected mice.

To determine if regions exhibiting enrichment in H3k9me2 also display increased occupancy of *SATB2*, we conducted ChIP-qPCR analysis using the previously validated antibody [53–55]. As a positive control, we assayed the enrichment of H3K9me2 and *SATB2* at the *SOX5* binding site within enhancer 434, upstream of the gene encoding *Fezf2* [53, 56]. Although we identified both H3K9me2 and *SATB2* within this region, neither displayed increased occupancy in EtOH-exposed affected samples (Fig. 6D). Further, although we could not identify *SATB2* binding at any candidate gene regulatory regions, we did detect occupancy of *SATB2* at repetitive sequences, and EtOH-exposed cortices displayed increased enrichment compared to controls (Fig. 6E). However, these primer sequences bind multiple genomic regions, and we cannot know if these sequences are common between H3K9me2 and *SATB2*. From these data, we conclude that although EtOH-exposed affected samples exhibit upregulation of the mammalian homolog of *DVE*-1, *SATB2* does not bind the regulatory regions of any of our candidate genes nor strictly correlate with all instances of increased H3K9me2 enrichment.

## Discussion

In this study, we found that alcohol-exposed tissues isolated from fetuses exhibiting structural defects exhibit disruptions in the genetic pathways controlling cellular respiration and oxidative phosphorylation, the majority of which are regulated by mTORC2. Although acute alcohol exposure is known to induce *mTORC2* activation in the CNS [57], our study is the first to identify prolonged programming of this complex, long after the removal of the toxicant. In glioblastoma multiforme, the acetyl-CoA-dependent acetylation of *RICTOR*, a core component of the *mTORC2* signaling complex, promotes an autoactivation loop, even when the upstream growth factor driving this signaling pathway is removed [58, 59]. Prenatal alcohol exposure associates with the *ACSS2*-dependent formation of acetate within the gestating brain [60]. Consistent with this study and adult models of alcohol exposure [3], we also observe transient increases in H3K9 acetylation but only during the exposure phase and not after alcohol is removed [12]. As increased acetate drives increased cellular levels of acetyl-CoA, we speculate that during gestation, binge exposures program an acetate-driven increase in *mTORC2* activity, resulting in a lasting suppression of the genetic pathways driving cellular respiration and the oxidative stress response. Additional experiments are required to determine if prenatal alcohol exposure is associated with the acetyl-CoA-dependent acetylation of *RICTOR*.

Consistent with our previous in vitro studies [12] and prior publications that examine chronic models of adult alcohol exposure [61], our RNA-seq analysis of the affected transcriptome revealed a broad suppression of transcriptional pathways that counter oxidative stress, while alcohol-exposed unaffected tissues do not. Similar to adult tissues, these signatures of differential gene expression and altered H3K9me2 are tissue-specific [62]. Interestingly, in our model, female offspring appear more prone to dysregulation of these pathways and overt dysgenesis. These data are consistent with recent studies indicating female offspring are more susceptible to the teratogenic impacts of intrauterine alcohol exposures [63]. Future studies are needed to determine if this mTORC2 signature is present in other models of exposure, is sex-specific, and if pharmacological interventions that target this pathway rescue EtOH-induced patterning defects.

In our model, affected tissues consistently display widespread increases in the enrichment of H3K9me2, which appear in genic, repetitive, and non-transcribed regions of the genome. Despite an established role of H3K9me2 in the formation of heterochromatin [19], we do not observe widespread silencing of gene expression. As specific examples, we previously reported a 500% increase in the enrichment of H3K9me2 at both the *Nkx2.1* and *Wnt5b* promoters [12]. Notwithstanding this dramatic increase, we find that the expression of both of these genes increased in alcohol-exposed affected cortices. In contrast, the other 14 H3K9me2-enriched candidate genes we previously identified did not display any significant changes in gene expression. Further, despite the widespread increase in H3K9me2, our RNA-seq analysis identified a near equivalent number of candidate genes that were up- and down-regulated. Although we acknowledge that increased H3K9me2 may correlate with the suppression of genes we did not assay here, the fact that some of our top upregulated candidate genes exhibited increased enrichment and that we observed augmented H3K9me2 in a non-transcribed region of the genome, we conclude that this change in chromatin structure does not always silence gene expression. Importantly, these observations are consistent between both our mouse and cell culture models.

Although H3K9 methylation predominantly associates with transcriptional silencing, similar to DNA methylation, this modification is also found in gene bodies and associates with transcriptional elongation [64–66]. Further, microscopy-based studies employing FISH have revealed that while deposition of H3K9me2 correlates with the localization of gene sequences into large domains at the nuclear periphery, this does not always associate with transcriptional silencing [67–70]. As an example, during both muscle and T-cell development, key homeobox genes driving progressive changes in cell identity need to be released from the nuclear periphery in order to achieve optimal transcriptional activation. However, most genes moving to or from the nuclear periphery display little to no change in expression, despite dynamic alterations in H3K9me3 [71–73]. Importantly, if a gene locus that becomes relocated to the nuclear periphery and silenced during differentiation is artificially moved to the nuclear periphery outside of a differentiation context, it does not become silenced [72, 74]. Similarly, although CAS9-mediated increases in H3K9 methylation across a large region of human chromosome 19 can induce the formation of heterochromatin-like domains, this large-scale change in chromatin structure did not result in widespread gene repression [20]. From these various studies, it appears that although increased H3K9 methylation may associate with transcriptional suppression, direct regulation of gene expression falls to protein factors (activators/repressors) located in the various nuclear compartments during specific developmental windows; not by a change in histone methylation. Given the association between H3K9 methylation and DNA methylation [75], future studies will determine if DNA methylation changes also associate with dysgenesis of the fetal cortex.

The induction of *Satb2* in the cortices of EtOH-exposed affected offspring, combined with the disruption of the transcriptional programs regulating mitochondrial function, prompted us to consider that similar to worms [17], mammalian cells also induce a chromatin-based stress response to stabilize nuclear interactions. Consistent with this idea, in osteoblasts, *SATB2* messenger RNA and protein upregulate in response to oxidative stress, and loss of *SATB2* disrupts chromatin structure and causes nuclear damage [76]. *SATB2* interacts with the H3K9me2 methyltransferase *EHMT2* (*G9a*), which promotes the association of target loci with the nuclear lamina [67, 77, 78]. However, outside of transposable elements and repetitive sequences, we could not identify *SATB2* binding at any candidate region exhibiting altered expression or increased H3K9me2. Thus, from these data, we conclude that although gestational alcohol exposure may induce *SATB2*, this protein does not appear to drive the altered H3K9me2 or transcriptional changes observed in EtOH-exposed affected tissues.

## Conclusions

One of the major confounding elements in the study of fetal alcohol spectrum disorders (FASDs) is the enormous variation observed in phenotypes and incidence [79]. FASD-associated defects have a wide range of severity, and while many women who drink have affected children, a significant subset of alcohol-exposed babies remain unaffected [80]. However, the vast majority of animal model-based studies have ignored this variation and have contrasted measures between control and alcohol-exposed tissues. Thus, we have a very poor understanding of what molecular changes distinguish affected versus unaffected individuals. Our studies indicate that changes in histone H3 lysine 9 dimethylation associate with alcohol-induced defects but that this epigenetic change may not directly influence the transcriptional programs driving development.

## Supplementary Information

**Additional file 1.** Primer sequences used in this study.

## Data Availability

The datasets generated and/or analyzed during the current study are available in the Gene Expression Omnibus (GEO) repository, GSE163300.

## Abbreviations

FASD: Fetal alcohol spectrum disorders
HDAC: Histone deacetylase
H3K9me2: Dimethylated histone H3, lysine 9
H3K9me3: Trimethylated histone H3, lysine 9
H4K20me3: Trimethylated histone H4, lysine 20
mTORC2: Mammalian target of rapamycin complex 2
DVE: Defective proVEntriculus
Satb1: Special AT-Rich Sequence Binding Protein 1
Satb2: Special AT-Rich Sequence Binding Protein 2
Ehmt2 (G9a): Euchromatic Histone Lysine Methyltransferase 2
Acss2: Acyl-CoA Synthetase Short Chain Family Member 2
Atp5e: ATP Synthase F1 Subunit Epsilon
Atp5mf: ATP Synthase Membrane Subunit F
Atp5l: ATP Synthase Membrane Subunit G
Ascl1: Achaete-Scute Family BHLH Transcription Factor 1
Cox7b: Cytochrome C Oxidase Subunit 7B
Dlx2: Distal-Less Homeobox 2
EIF2: Eukaryotic Translation Initiation Factor 2A
Ndufa7: NADH dehydrogenase [ubiquinone] 1 alpha subcomplex subunit 7
Nkx2.1: NK2 Homeobox 1
IAP: Intracisternal A-particle
LINE: Long interspersed nuclear elements
Untr6: Untranscribed region of chromosome 6
Fezf2: FEZ Family Zinc Finger 2
